# 1054. Activity of a Anti-staphylococcal Lysin, LSVT-1701: *In vitro* Susceptibility of *Staphylococcus aureus* and Coagulase-Negative Staphylococci (CoNS) Global Clinical Isolates (2002 to 2019)

**DOI:** 10.1093/ofid/ofab466.1248

**Published:** 2021-12-04

**Authors:** David Huang, Helio S Sader, Paul R Rhomberg, Katyna Borroto-Esoda, Eric Gaukel

**Affiliations:** 1 Lysovant, Houston, Texas; 2 JMI Laboratories, North Liberty, Iowa

## Abstract

**Background:**

LSVT-1701, formerly SAL200, is a novel, recombinantly-produced, bacteriophage-encoded lysin that specifically targets staphylococci via cell wall enzymatic hydrolysis. We reported the *in vitro* activity of LSVT-1701 against clinical isolates of *S. aureus* and coagulase-negative staphylococci (CoNS) collected worldwide.

**Methods:**

LSVT-1701 and comparators were tested against 415 *S. aureus* (n=315) and CoNS (n=100) clinical isolates expressing various resistance phenotypes. The isolates were collected in 2002-2019 from medical centers located in the United States (50 medical centers; 174 isolates; 41.9% overall), Europe (37 medical centers; 140 isolates; 33.7% overall), Asia-Pacific region (15 medical centers; 55 isolates; 13.3% overall), and Latin America (12 medical centers; 46 isolates; 11.1% overall). These isolates originated mostly from the year 2019 (n=323).The isolates were susceptibility tested by the CLSI broth microdilution method. MIC interpretations were based on CLSI and EUCAST criteria where available.

**Results:**

LSVT-1701 was highly active against *S. aureus* and CoNS isolates with MIC_90_ values of 2 mg/L for all *S. aureus*, methicillin-susceptible *S. aureus* (MSSA), methicillin-resistant *S. aureus* (MRSA), and CoNS (Table). The highest LSVT-1701 MIC values were 4 and 8 mg/L among *S. aureus* and CoNS, respectively. LSVT-1701 retained potent activity against *S. aureus* isolates showing resistance or decreased susceptibility to oxacillin, vancomycin, teicoplanin, telavancin, linezolid, daptomycin, ceftaroline, or lefamulin; MIC50 values ranged from 0.5 to 1 mg/L and MIC_90_ values ranged from 1 to 4 mg/L among *S. aureus* resistant subsets.

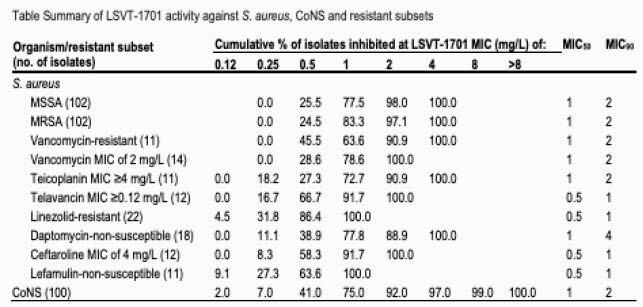

Summary of LSVT-1701 activity against S. aureus, CoNS and resistant subsets

**Conclusion:**

LSVT-1701 demonstrated potent *in vitro* activity against contemporary clinical isolates of *S. aureus* and CoNS collected from medical centers worldwide and against resistant *S. aureus* isolates with uncommon resistance phenotypes. The results of this study support further clinical development of LSVT-1701 to treat staphylococcal infections.

**Disclosures:**

**David Huang, MD, PhD**, **Lysovant** (Consultant) **Helio S. Sader, MD, PhD, FIDSA**, **AbbVie (formerly Allergan**) (Research Grant or Support)**Basilea Pharmaceutica International, Ltd.** (Research Grant or Support)**Cipla Therapeutics** (Research Grant or Support)**Cipla USA Inc.** (Research Grant or Support)**Department of Health and Human Services** (Research Grant or Support, Contract no. HHSO100201600002C)**Melinta Therapeutics, LLC** (Research Grant or Support)**Nabriva Therapeutics** (Research Grant or Support)**Pfizer, Inc.** (Research Grant or Support)**Shionogi** (Research Grant or Support)**Spero Therapeutics** (Research Grant or Support) **Paul R Rhomberg**, **Cidara Therapeutics, Inc.** (Research Grant or Support)**Pfizer, Inc.** (Research Grant or Support) **Katyna Borroto-Esoda, PhD**, **Lysovant** (Consultant) **Eric Gaukel, BS**, **Lysovant** (Employee)

